# Understanding the Needs of a Mobile Phone–Based Telemonitoring Program for Pregnant Women at High Risk for Pre-Eclampsia: Interpretive Qualitative Description Study

**DOI:** 10.2196/32428

**Published:** 2022-02-24

**Authors:** Anam Shahil Feroz, Kristina De Vera, Nadia D Bragagnolo, Sarah Saleem, Zulfiqar Bhutta, Emily Seto

**Affiliations:** 1 Institute of Health Policy, Management, and Evaluation University of Toronto Toronto, ON Canada; 2 Community Health Sciences Department Aga Khan University Karachi Pakistan; 3 Physical Sciences Sunnybrook Health Sciences Centre Toronto, ON Canada; 4 Centre for Global Child Health SickKids Toronto, ON Canada; 5 Center of Excellence in Women and Child Health Aga Khan University Karachi Pakistan; 6 Dalla Lana School of Public Health University of Toronto Toronto, ON Canada; 7 Centre for Global eHealth Innovation, Techna Institute University Health Network Toronto, ON Canada

**Keywords:** telemonitoring, pre-eclampsia, qualitative study, Pakistan, pregnant women at high risk, low- to middle-income country, pregnant, pregnancy, women, mobile phone

## Abstract

**Background:**

Lack of early risk detection, diagnosis, and treatment of pregnant women at high risk for pre-eclampsia can result in high maternal mortality and morbidity not only in Pakistan but also in other low- to middle-income countries (LMICs). A potential tool for supporting pregnant women at high risk for pre-eclampsia for early detection is telemonitoring (TM). However, there is a limited body of evidence on end-user needs and preferences to inform the design of the TM programs for pregnant women at high risk for pre-eclampsia, specifically in LMICs such as Pakistan.

**Objective:**

This study aims to explore the needs of TM for pregnant women at high risk for pre-eclampsia in Karachi, Pakistan, to inform a potential future feasibility trial of a mobile phone–based TM program.

**Methods:**

An interpretive qualitative description approach was used to conduct and analyze 36 semistructured interviews with 15 (42%) pregnant women and 21 (58%) key informants, including clinicians; nurses; maternal, neonatal, and child health specialists; and digital health experts to explore the perspectives, needs, and preferences of a mobile phone–based TM program to support pregnant women at high risk for pre-eclampsia. Pregnant women were identified through heterogeneous sampling, whereas key informants were selected through purposive sampling. The interview transcripts were analyzed using a conventional content analysis technique.

**Results:**

The following four themes emerged from the analysis of the transcripts: poor use of antenatal care during pregnancy, the value of a TM program in high-risk pregnancy, barriers influencing the adoption of TM programs and potential strategies, and considerations for implementing TM programs. The pregnant women and health care providers were willing to use a TM program as they perceived many benefits, including early identification of pregnancy complications, prompt treatment, convenience, cost-effectiveness, increased sense of empowerment for one’s health care, improved care continuity, and reduced clinical workload. However, some providers and pregnant women mentioned some concerns regarding the adoption of a TM program, including malfunctioning and safety concerns, potential inaccuracy of blood pressure machines, increased clinical workload, and resistance to learning new technology. Our study recommends building the capacity of patients and providers on TM program use, sensitizing the community and family members on the usefulness of the TM program, using an approach incorporating user-centered design and phased implementation to determine the clinical workload and whether additional staff for the TM program is required, and ensuring greater levels of co-design and the engagement of consumer representatives.

**Conclusions:**

Our findings highlight the perceived feasibility of a mobile phone–based TM program for pregnant women at high risk for pre-eclampsia and provide insights that can be directly used for the design of future TM programs with the aim of reducing mortality and morbidity from pre-eclampsia and eclampsia in LMICs.

## Introduction

### Background

In Pakistan, approximately one-third (34%) of maternal mortality in tertiary-level facilities is attributable to pre-eclampsia and eclampsia [1]. High maternal mortality from pre-eclampsia and eclampsia results from the lack of early risk detection, diagnosis, and treatment of pregnant women at high risk for pre-eclampsia [1]. To meet the United Nations Sustainable Developmental Goal target of 3.1 (maternal mortality ratio <70/100,000 live births) by 2030 [2], innovations are required to help decrease pre-eclampsia and eclampsia related mortality. There is substantial empirical evidence on the use of telemonitoring (TM) to support pregnant women at high risk for pre-eclampsia by remotely monitoring their blood pressure readings at home [3-11]. TM is a promising tool in which individuals who are pregnant take blood pressure measurements and record symptoms at home, and these readings and self-reported symptoms are sent to their health care providers in real time [9]. Van Den Heuvel et al [10] found that pregnant women at high risk in the Netherlands could highly benefit from TM as it facilitates better blood pressure control, early risk identification and treatment, fewer hospital visits, and cost savings [10]. In Pakistan, TM has been implemented by community health workers as part of the Community-Level Interventions for Pre-eclampsia (CLIP) trial [9] and Control of Blood Pressure and Risk Attenuation–Bangladesh, Pakistan, and Sri Lanka studies [12]. In the CLIP trial, the Piers on the Move mobile health app directed community health workers to first observe women to rule out emergency conditions that would warrant immediate referral to a facility. The CLIP Piers on the Move tool facilitated the stratification of pregnant women by community health workers into 1 of 3 care pathways: usual antenatal and postnatal care, nonurgent referral, and urgent referral to a higher facility. The CLIP trial was well received by families; however, it did not have a significant impact on either the composite outcome of maternal, fetal, and newborn mortality and severe morbidity or individual components thereof [13].

Although the literature exploring the use of TM for supporting pregnancy care is expanding [10,14-16], there are limitations in the data collected from TM systems, such as monitoring of only a few gestation parameters, which makes it hard or impossible for health professionals to provide holistic assistance to the pregnant women and fetuses [16]. The review by Eysenbach et al [17] on the effectiveness of TM in obstetrics concluded that TM could be tentatively recommended for pregnant women at risk for preterm delivery, given the high methodological risk of bias among the included studies [18]. In addition, very few studies have focused on understanding patient needs for the design and development of TM platforms. The limited body of qualitative evidence on end user needs and preferences to inform the design of TM programs could be a barrier to the successful development and implementation of more applicable, effective, and user-centric TM platforms [19-22]. A qualitative study conducted at Vanderbilt University Medical Center explored the practices, health needs, and strategies related to pregnancy care for pregnant women and caregivers to inform the development and implementation of health information technologies [23]. Most expectant mothers in the study encountered everyday problems with mobility and household management and desired more assistance from caregivers, who often did not know how to help. The study identified technological innovations, including health-tracking watches to take basic vital measurements, virtual assistants, and cellular apps, to connect fellow pregnant women with others in their region to support expectant families [22].

Most reported TM programs had been implemented in high-income countries (eg, United Kingdom, Canada, United States, and Belgium) [6,7,9,10], with a paucity of evidence on the use of TM to support pregnant women at high risk in low- to middle-income countries (LMICs). In our scoping review on the use of digital health interventions for pregnant women at high risk for pre-eclampsia in LMICs, we identified only 9 unique digital health interventions from mainly South Asia and sub-Saharan Africa. Of these interventions, 2 served the purpose of predicting risk for adverse maternal health outcomes, whereas 7 focused on monitoring pregnant women at high risk, for managing pre-eclampsia and eclampsia (publication in review). The review identified only 1 TM intervention for monitoring pre-eclampsia and eclampsia in an LMIC. This was the Pre-eclampsia Integrated Estimate of RiSk on the Move app, which was used in the CLIP trials in India, Pakistan, and Mozambique, conducted from 2014 to 2017.

### Objective

TM is a complex intervention and is sensitive to the context in which it is applied, including sociodemographic and sociocultural considerations, financial constraints, clinical workflows, and health system systems [24]. Thus, it cannot be assumed that the TM needs of pregnant women in high-income countries will necessarily apply to pregnant women in LMICs. We propose to assess the feasibility of a mobile phone–based TM program to support pregnant women at high risk for pre-eclampsia at Jinnah Post Graduate Medical Center (JPMC) in Karachi, which is the largest city in Pakistan. As the first step of this project, this study aims to explore the needs of TM for pregnant women at high risk for pre-eclampsia in Karachi, Pakistan, to inform a potential future feasibility trial of a mobile phone–based TM.

## Methods

### Research Design and Setting Overview

Given our intention to understand the local context and needs of the intended users, an interpretive descriptive design was used [25] to explore the perspectives, needs, and preferences of pregnant women at high risk for pre-eclampsia for mobile phone–based TM through semistructured interviews with patient participants and key informants. Patient participants included pregnant women at high risk for pre-eclampsia who were the intended beneficiaries of the TM program, whereas key informants included the diverse group of stakeholders who provide or supply health care in different capacities, including nurses; clinicians; maternal, neonatal, and child health (MNCH) specialists; and digital health experts. This study was conducted at the JPMC, a 1650-bed tertiary-level public sector hospital in Karachi, which provides hospital care to >1 million people of low socioeconomic status coming from Karachi, Interior Sindh, Baluchistan, and other remote areas [1]. This research focused on the outpatient area of the JPMC obstetrics and gynecology (OB-GYN) department, which serves the vast majority of low-income women with high-risk pregnancies.

Further details about the study sites and protocol have been previously published [19]. The study was approved by the Aga Khan University (AKU) ethical review committee (2020-2153-8519), the JPMC institutional review board (44379), and the University of Toronto research ethics board (30635).

### Proposed Mobile Phone–Based TM Program

During the interviews, patient participants were provided with an overview of the proposed mobile phone-based TM program. The proposed TM program includes the use of a Bluetooth-enabled home blood pressure device that is validated for use during pregnancy and a mobile app (in the Urdu language). The TM program will enable pregnant women to take their blood pressure reading every morning at home and answer symptom questions using the mobile app. All enrolled women would receive real-time automated instructions based on their readings, such as taking additional blood pressure readings, calling the medical officer (ie, a trained physician in Pakistan), or visiting the OB-GYN emergency department. The real-time automated instructions would be delivered in Urdu text on the mobile app. The medical officer would receive alerts from the TM system if their patient’s blood pressure values were out of the target range. The medical officer would act as a central point person to communicate with the patients (phone calls or using the asynchronous app chat feature) and with the rest of the participant’s care team as needed.

### Participant Recruitment and Eligibility Criteria

The interpretive description approach used a maximum variation sampling technique [26,27] to purposively recruit a range of pregnant women at high risk for pre-eclampsia who had differing needs and preferences for the mobile phone–based TM program. Textbox 1 provides the definition of pregnant women at high risk for pre-eclampsia as per the National Institute for Health and Care Excellence guidelines. Owing to the COVID-19 pandemic–related restrictions, nurses at the hospital were asked to support the identification and recruitment of eligible pregnant women at high risk for pre-eclampsia for in-person interviews. Nurses identified the eligible pregnant women from the JPMC outpatient department, where women wait for several hours to be seen by their health care providers. The nurses contacted the research staff once a potential participant was identified, and then, the research staff made an immediate visit to the clinic to conduct in-person patient interviews at the hospital. The key informants such as clinicians, including specialists in OB-GYN, nurses, MNCH specialists, and digital health experts, were purposively recruited from the JPMC OB-GYN department, AKU, and other leading health care institutions in Karachi, Pakistan. Textbox 2 provides a list of the eligibility criteria for the patient participants and the key informants.

**Definition of pregnant women at high risk for pre-eclampsia as per the National Institute for Clinical Excellence guidelines.**

**Definition**

National Institute for Health and Care Excellence guidelines define pregnant women at high risk for pre-eclampsia as those who have 1 high-risk factor or >1 moderate risk factor for pre-eclampsia.

**High-risk factors:**

- Hypertensive disease in a previous pregnancy
- Chronic kidney disease
- Autoimmune diseases, such as systemic lupus erythematosus or antiphospholipid syndrome
- Type 1 or type 2 diabetes
- Chronic hypertension

**Moderate risk factors:**

- First pregnancy
- Aged ≥40 years
- Pregnancy interval of >10 years
- BMI of ≥35 kg/m^2^ at the first visit
- Family history of pre-eclampsia
- Multifetal pregnancy

**Eligibility criteria of patient participants and key informants.**

**Pregnant women at high risk for pre-eclampsia**

**Inclusion criteria:**

- Pregnant women at high risk for pre-eclampsia who were visiting the Jinnah Post Graduate Medical Center outpatient department for antenatal visits
- Pregnant women at high risk for pre-eclampsia who met the National Institute for Health and Care Excellence guidelines [27] definition of high risk for pre-eclampsia
- Pregnant women who were diagnosed as high risk for pre-eclampsia for at least 3 weeks and have had time to reflect on the disease condition, associated difficulties, and needs for telemonitoring
- Pregnant women with the ability to speak English, Urdu, or Sindhi language

**Exclusion criteria:**

- Pregnant women at high risk for pre-eclampsia who were admitted to the inpatient wards and emergency care for treatment purposes
- Pregnant women at high risk for pre-eclampsia who refuse to consent for participating in the needs assessment study

**Key informants**

**Inclusion criteria:**

- Key informants must be clinicians; nurses; maternal, neonatal, and child health specialists; or digital health experts who were directly or indirectly involved in the care of pregnant women at high risk for pre-eclampsia
- Key informants with the ability to speak Urdu or English language

**Exclusion criteria:**

- Key informants who refuse to consent for participating in the needs assessment study

### Data Collection Methods

Interviews were conducted between March 2020 and August 2020. The research team and study participants had access to all necessary personal protective equipment to help prevent the risk of spreading the COVID-19 virus during data collection. Midwives and nurses at JPMC used the National Institute for Health and Care Excellence guidelines [28] to identify pregnant women at high risk for pre-eclampsia from the outpatient department of the JPMC hospital. The identified pregnant women were informed about the study’s purpose and procedures (including the recording of interviews), and their willingness to participate in the study was ascertained by the primary researcher (ASF). If the participant was unable to read the consent form, the primary researcher explained the consent form verbally in their local language. Pregnant women at high risk for pre-eclampsia who were unable to write their names were asked to provide a thumbprint to mark their consent to participate. The interviews were conducted on the same day by the primary researcher when eligible pregnant women were identified. To avoid any disruption during the interview and ensure confidentiality, the consenting pregnant women were asked to move to a separate private room for the interview. We anticipated conducting and recording 10 to 15 interviews with pregnant women at high risk, to reach data saturation. The intent was to allow caregivers (husband, mother-in-law, or any family member) to accompany the pregnant women during the interviews if preferred by the patient. However, caregiver involvement during the interview was not possible because of COVID-19–related restrictions. After the interviews, the patient participants were given a 10-minute illustrative presentation to provide them with an understanding of the pre-eclampsia condition, its consequences, and simple ways to manage the condition through regular follow-ups. Key informants such as clinicians, nurses, MNCH specialists, and digital health experts were interviewed to understand their perspectives, preferences, and needs regarding the use of TM for pregnant women at high risk for pre-eclampsia. The key informants were identified from AKU, JPMC, People’s Primary Healthcare Initiative, Sehat Kahani, Digital Care, eHealth Association of Pakistan, Commission on Science and Technology for Sustainable Development in the South, Shaheed Zulfikar Ali Bhutto Institute of Science and Technology, Tech4Life Enterprises, the Aga Khan Development Network Digital Health Resource Center, and other relevant institutions. All the key informants were invited to participate in the qualitative study via email, and key informants were requested to sign informed consent forms before the interview began. Most interviews with key informants were conducted through the web via Zoom (Zoom Video Communications, Inc) in either Urdu or English, whereas a few interviews with clinicians and nurses were conducted face to face at the JPMC OB-GYN department. We anticipated conducting 13 to 15 interviews with key informants to reach data saturation. The interviews lasted between 40 and 60 minutes.

The approach by Spradley [29] was used to design 2 semistructured interview guides for pregnant women at high risk for pre-eclampsia and key informants. This approach emphasizes the importance of having *grand tour* questions to allow for the free flow of rich and deep information. The interview guide for pregnant women at high risk for pre-eclampsia involved a general discussion about pre-eclampsia, causes of pre-eclampsia, perceptions toward the use of TM for pregnant women at high risk for pre-eclampsia, perceived benefits of TM, potential limitations or concerns related to TM for pre-eclampsia, and feasibility of smartphone-based TM. The interview guide for key informants included grand questions on causes of pre-eclampsia, routine obstetric care for pre-eclampsia, use of TM for supporting pregnant women at high risk for pre-eclampsia, and perceived facilitators of and barriers to the implementation of TM. The guide was pilot-tested with 2 pregnant women at high risk for pre-eclampsia and 2 key informants who shared the same traits as the study sample [30,31].

### Data Analysis

The audio recordings from the interviews were professionally transcribed and translated into the English language, with no identifying characteristics included in the transcriptions. The anonymized transcripts were uploaded to NVivo (version 12 Plus; QSR International) to enable easy and organized retrieval of data for analysis. The conventional content analysis approach [32] was used to inductively analyze all the interview transcripts. The primary researcher (ASF) independently coded all the transcripts as the primary reviewer, whereas KDV and NDB independently coded key informants’ and patients’ interviews, respectively, as the second reviewers. The interviews of key informants and patient participants were analyzed separately by the researchers, and later, the codes and themes were compared across the 2 groups to identify overarching themes and subthemes. The main themes and subthemes were identified independently by the primary researcher and second reviewers for the 2 groups of respondents and then discussed in the larger research group until agreement on the themes was achieved. To gain a more complete understanding of the perspectives, preferences, and needs of TM for women at high risk for pre-eclampsia, the subthemes from all the interviews were compared and contrasted by multiple researchers (ASF, KDV, and NDB) to seek convergence and corroboration through data triangulation between the patient and key informant interviews [27,33]. The four main themes, which were consistent between the patient and key informant groups, and subthemes were finalized once a consensus was achieved after a total of four meetings: 2 meetings with the group of reviewers who analyzed the transcripts and 2 meetings with the larger research group.

## Results

### Overview

A total of 36 semistructured interviews were conducted to explore the perspectives, needs, and preferences of a mobile phone–based TM program to support pregnant women at high risk for pre-eclampsia. Of the 36 interviews, 15 (42%) interviews were conducted with pregnant women at high risk for pre-eclampsia, whereas 21 (58%) were conducted with various key informants, including 8 (38%) clinicians, 3 (14%) nurses, 3 (14%) MNCH specialists, and 7 (33%) digital health experts. Each of the interviews lasted between 30 and 50 minutes. All the participants (36/36, 100%) who were approached by the study team agreed to participate. The demographic information for all the key informants and patient participants is illustrated in Tables 1 and 2, respectively.

On the basis of the inductive analysis, four overarching themes were identified: (1) poor use of antenatal care during pregnancy, (2) value of a TM program in high-risk pregnancy, (3) barriers influencing adoption of TM and potential strategies, and (4) considerations for implementing TM programs. These themes and their subthemes are summarized in Textbox 3.

**Table 1:** Characteristics of key informants (N=21).

Characteristics and category			Values
**Gender, n (%)**			
	Female	16 (76)	
	Male	5 (24)	
**Age (years)**			
	Values, mean (SD)	46.21 (11)	
	Values, median (range)	47 (28-65)	
**Role, n (%)**			
	Nurses	3 (14)	
	Clinicians (OB-GYN^a^)	8 (38)	
	MNCH^b^ specialists and digital health experts	10 (48)	
**Specialty, n (%)**			
	OB-GYN	11 (52)	
	MNCH	3 (14)	
	Digital health	7 (33)	
**Experience (years)**			
	Values, mean (SD)	16.52 (10)	
	Values, median (range)	20 (4-40)	

^a^OB-GYN: obstetrics and gynecology.

^b^MNCH: maternal, neonatal, and child health.

**Table 2:** Characteristics of patient participants (N=15).

Characteristics of pregnant women at high risk for pre-eclampsia and category		Values
**Gender,** **n (%)**		
	Female	15 (100)
**Age** **(years)**		
	Values, mean (SD)	28.26 (5.284)
	Values, median (range)	28 (20-38)
**Educational level, n (%)**		
	No education	3 (20)
	Less than high school	3 (20)
	High school	8 (53)
	College or university	1 (7)
**Occupation, n (%)**		
	Housewife	11 (73)
	Professional	4 (27)
**History of pre-eclampsia, n (%)**		
	Yes	9 (60)
	No	6 (40)
**Pregnancy (weeks)**		
	Values, mean (SD)	31.67 (6.06)
	Values, median (range)	33 (12-37)
**Frequency of blood pressure measurement, n (%)**		
	Daily	5 (33)
	Once or twice a week	4 (27)
	Thrice a week	2 (13)
	As per need	3 (20)
	Never	1 (7)
**Access to a personal home blood pressure machine, n (%)**		
	Yes	2 (13)
	No	13 (87)
**Gravida^a^, n (%)**		
	Primigravida	4 (27)
	Multigravida	11 (73)
**Parity^b^, n (%)**		
	Nulliparity	4 (27)
	Multiparity	9 (60)
	Grand parity	2 (13)
**Access to a mobile phone, n (%)**		
	Basic mobile phone	4 (27)
	Smartphone	11 (73)
**Personal or shared access to a mobile phone, n (%)**		
	Individual access	9 (60)
	Shared access	6 (40)
**Access to the internet, n (%)**		
	Yes	8 (53)
	No	7 (47)

^a^Total number of pregnancies.

^b^Live births and stillbirths.

**Themes and categories.**

**Poor use of antenatal care during pregnancy**

- Inadequate access to quality maternal health care services
- Poor awareness and self-management during a high-risk pregnancy

**Value of a telemonitoring (TM) program in high-risk pregnancy**

- Early identification of pregnancy complications and prompt treatment
- Impact on physician’s workload
- Convenient and cost-effective
- Sense of empowerment in own health care

**Barriers influencing adoption of TM and potential strategies**

- Lack of willingness by pregnant women and health care providers to use TM program
- Weak technological literacy to use TM program
- Lack of technological infrastructure to use TM program
- Sociocultural factors impacting TM program use

**Considerations for implementing the TM program**

- Features and ease of use of TM program
- Handling, maintenance, and sustainability of the TM program

### Poor Use of Antenatal Care During Pregnancy

#### Inadequate Access to Quality Maternal Health Care Services

When asked about the use of antenatal care during current and previous pregnancies, some pregnant women described the accessibility issues they face in using antenatal care, such as the long commute to the public hospital. They reiterated that traveling is time consuming and costly, given that the public hospitals are few and quite far from their residential areas:

Every time I had to spend Rs. 1000 for JPMC visit. Obviously, this [per visit costs for travel] is very hard for us to afford.

Woman with pregnancy 03

Most pregnant women at high risk for pre-eclampsia mentioned that they usually visit small private clinics near their residential areas to receive care in the first few months of their pregnancy. However, these clinics are not well-equipped and are capable of managing only normal pregnancies. For this reason, pregnant women at high risk are referred to a tertiary-level public hospital such as JPMC by these private clinics for management and hospital delivery:

Before this [JPMC], I was going to a private hospital at Korangi's side for checkups. They [Doctor at the private hospital] told me that maybe there is some issue with the umbilical cord, so, maybe you would need to be operated...and referred me to Jinnah. So, I made my antenatal card over here [JPMC].

Woman with pregnancy 12

Most pregnant women at high risk described an unpleasant environment at the public hospital because of the excessive environmental noise, crowds, and long waiting times:

Actually, we have to wait a lot here and the atmosphere is a little supportive...the people [pregnant women] who come here [for antenatal care] have to wait for most of the time...and the noise here [due to crowd] makes us go into more depression. So, this is a bit of an issue.

Woman with pregnancy 05

Key informants highlighted the impact of high physician workload on the quality of patient care and health education, especially in public sector hospitals. Owing to the increased volume of patients and time constraints, providers often found it difficult to educate pregnant women at high risk about self-management such as dietary advice, regular blood pressure monitoring, regular antenatal visits, and the importance of physical activity and exercise during pregnancy. In addition, the increased workload of health care workers was thought to not enable providers to counsel pregnant women about the adverse outcomes of the disease condition:

You know our physicians are overwhelmed, they do not have time or even the realization that they need to educate their patients. So, it begins from there.

Digital health expert 14

#### Poor Awareness and Self-management During a High-Risk Pregnancy

The interviews with pregnant women and key informants revealed that pregnant women have poor awareness and knowledge about pre-eclampsia and eclampsia, its symptoms, and pregnancy care in general. A few pregnant women stated that they knew about the disease symptoms but only through their current and previous pregnancy experiences. Key informants highlighted that pregnant women generally do not understand their disease condition because of low literacy levels, and when they begin to realize and report the disease symptoms, they get tagged as normal pregnancy symptoms:

Women do not even know what their symptoms are, so they are generally not educated enough to understand what they are going through...So, lots of these women do not even know what is happening, and by the time they do start realizing, it is routinely discarded off just as the symptoms of the pregnancy, it is not investigated further.

Digital health expert 02

The interviews revealed that inadequate awareness about the disease condition influences the self-care behaviors of pregnant women, such as dietary precautions, regular exercise, and blood pressure monitoring among pregnant women at high risk. When asked about self-care behaviors during pregnancy, most pregnant women described the different types of dietary precautions they take during pregnancy. However, none of them reported performing exercise and physical activity during pregnancy. In addition, pregnant women reported measuring their blood pressure during pregnancy either through a home blood pressure machine or visiting clinics. The frequency of blood pressure measurement varied among all pregnant women. A minority of pregnant women verbalized that daily blood pressure monitoring caused them stress, and therefore, they tend to avoid measuring blood pressure unless it is required.

In addition, pregnant women at high risk for pre-eclampsia and key informants described several sociocultural and financial factors leading to poor disease awareness and self-management. The pregnant women in the study received different levels of support from their husbands, in-laws, extended family members, and colleagues at the workplace for pregnancy care. Most pregnant women at high risk for pre-eclampsia acknowledged the support they received from their husbands and in-laws during pregnancy, whereas some pregnant women seemed to have trouble finding support for their responsibilities, especially childcare. Therefore, they did not have time to attend antenatal visits or get their blood pressure checked at a nearby clinic. Key informants further mentioned that women do not receive enough attention and care during the pregnancy period:

They [Pregnant women] have a great busy schedule at home. They cannot afford the luxury of enjoying downtime during their pregnancy they work really hard...their husbands and in-laws do not accept that these pregnant mothers need to have better hygiene, better nutrition, and rest hours. So, all of these factors contribute to poor pregnancy care and we all are aware of these factors, and there is nothing hidden.

Digital health expert 14

Key informants believed that cultural norms influence the empowerment of pregnant women to make decisions about their pregnancy care. They stated that women do not consider their health as a priority over other things, largely because of cultural factors. One such instance that key informants highlighted was that pregnant women do not make use of the health card provided to them by the public sector hospital upon registration of their pregnancy. The intent of the health card is for them to receive antenatal services; instead, the women would only visit the hospital and use their health card at the time of delivery or if there was an emergency:

So, they only have the card with them, and if you see, then there is only one entry on it...and on it is mentioned that you have to get all these tests done regularly but they never come. They only come at the time of delivery.

Clinician 03

Key informants described that pregnant women are empowered by health care workers during regular clinic visits to improve self-care behaviors. However, health care workers forget that the household decision-making power is with husbands and mothers-in-law. Thus, they suggested having husbands and family members be present along with pregnant women during health education sessions to have a significant impact on the uptake of maternal health services:

So, most of the time we try to empower the pregnant woman, and we forget that she does not have the decision-making power in that set. So, it is very important for us, to understand the dynamics, so the husband is very important. He needs to be on board and educated when it comes to the self-care of his wife. A mother-in-law is very important because in most cases she is the decision-maker. So, she needs to be very much aware of the situation and, also aware of what needs to be done and If that is not done, what will happen.

Digital health expert 11

In terms of financial constraints, some women expressed that they could not afford to buy nutritious food, medicine for their pre-eclampsia condition, or equipment for the regular monitoring of blood pressure at home. Most pregnant women acknowledged the importance of regular blood pressure monitoring. However, they mentioned that they could not get regular blood pressure measurements during their pregnancy as their husbands had to accompany them to the clinic, which often required their husbands to take time off from work and for the pregnant women to work around their husbands’ schedules.

### Value of a TM Program in High-Risk Pregnancy

#### Early Identification of Pregnancy Complications and Prompt Treatment

Approximately all key informants and most pregnant women at high risk for pre-eclampsia believed that the TM program would aid in the early identification of alarming signs through regular monitoring of blood pressure and disease symptoms. A few clinicians and MNCH specialists expressed that the TM program is highly valuable for reducing maternal and child morbidity and mortality rates:

Its benefit is that if even 90% of it is being done then it would be very beneficial. Meaning that the patients with us having eclampsia would not be dying. Because when they come to us in tertiary care. They come in bad to worse conditions...The mortality rate increases and morbidity itself is increased in those patients. And because of this our mortality and morbidity can be decreased.

Clinician 04

Key informants further mentioned that the early identification of danger signs would help in the timely referral of pregnant women at high risk to secondary health care facilities where their condition could be managed through either medications or immediate delivery. Although some key informants expressed that TM should be complementary to antenatal visits as clinicians would like to see pregnant women at high risk in clinics to monitor signs that cannot be captured by TM programs, an expert described an emergency where health care providers can immediately intervene with the help of the TM program:

So, if there are no kicks [fetal movement] for the last eight hours or ten hours we [healthcare providers] can immediately intervene and have the patients come to the hospital, in an emergency, do the ultrasound and do some kind of intervention to save the life of the baby. So, that why it is important. It is very, very important, especially for precious pregnancies.

Digital health expert 14

Pregnant women articulated that blood pressure readings would be more accurate when taken at home as they would not have to travel and wait for long hours in the stressful environment of JPMC to be seen by health care providers. In addition, pregnant women felt that there would be improved care continuity through this TM program as they will remain connected with their respective physicians through mobile phones without needing to go out of their homes:

There are many benefits like most doctors say that due to blood pressure anything can happen to the child and mother. Both can be saved. Through the TM program doctor will remain connected to us through the machine, phone message, or call...so it is much better. Then I would not have to go out of my home, I can stay at home and talk to my doctor.

Woman with pregnancy 02

#### Impact on Physician’s Workload

Key informants, including clinicians and nurses, believed that the TM program could help in reducing physicians’ workload and crowds in public hospitals, given that face-to-face visits by pregnant women would be reduced to a large extent. Although some key informants thought that this is a potential benefit of implementing a TM program, others argued that it might also be detrimental to clinicians’ time as clinicians would need to remotely monitor and track the health conditions of pregnant women through the TM program. In addition, key informants stated that being able to remotely monitor the health conditions of pregnant women would empower health care providers and facilitate better clinical decision-making for managing high-risk pregnancies. The experts thought that the TM program would simplify their work as the blood pressure monitoring data will flow regularly to health providers through the smartphones of pregnant women, which would help in clinical decision-making:

It simplifies their (healthcare provider) work it facilitates in monitoring their women on regular basis...it becomes very, very important to give these devices to women so that the data can flow regularly to these health providers...to help them make a better decision about the patients or the women that they are taking care of.

Digital health expert 14

#### Convenient and Cost-effective

Most pregnant women and key informants felt that the TM would be convenient as patients will be able to easily monitor their blood pressures at their homes. They further iterated that this would save them time and the costs they incur when visiting the public hospital to receive pregnancy care. Most importantly, pregnant women believed that there would be less disruption to their daily routine, and they would not have to depend on their husbands or in-laws for traveling to the hospital and for childcare responsibilities:

It would be quite beneficial as I have two children with me at home, whom I keep with someone when I come here (JPMC). Because my own mother cannot take care of them because she has a job, and my mother-in-law would not take care of them. So, for me, a blood pressure machine is beneficial in this way that I can give my record while staying at home and can be with my children too.

Woman with pregnancy 02

#### Sense of Empowerment in Own Health Care

Pregnant women at high risk for pre-eclampsia felt that they would be more confident about their health through the use of the TM program and would gain a sense of empowerment in their own health care decisions. Several key informants also articulated that the TM program might prove essential for increasing personal health responsibility, given that the program would encourage self-care behaviors among pregnant women. The act of monitoring, reporting blood pressures via phone, and receiving alerts and messages on appropriate self-care behaviors during high-risk pregnancy would, in turn, empower pregnant women at high risk:

Most important is women’s empowerment. Anything that we do in the communities to have the women monitor their own health, send information about their own self or receive information about how to better care about themselves...do[es] empower them within the families and in the communities and I think that is really important as well.

Digital health expert 09

### Barriers Influencing Adoption of TM and Potential Strategies

#### Lack of Willingness of Pregnant Women and Health Care Professionals to Use the TM Program

Some health care providers, including clinicians and nurses, were unwilling to use the TM program, given that it would require providers to perform additional tasks, whereas other health care providers showed a willingness to use the TM program and suggested implementing TM to leverage the high adoption of virtual care during the pandemic. Key informants expressed that health care providers would not readily accept the TM program, given that it will require them to invest time and effort to learn and use it:

So, I think the biggest problem is going to be that it will increase work for all the nurses and doctors...they already have so much work on their hands...trying any new gadget will increase time and will increase monitoring as well. So, I think one barrier will be, that they will not accept it readily because of the time being consumed in doing this.

Digital health expert 02

Although some key informants thought that implementing TM would lessen physician workload, most clinicians and nurses were reluctant to take complete responsibility for running the TM program at public sector hospitals, including regular and timely monitoring of blood pressure readings of pregnant women at high risk. Health care providers iterated that they are already overwhelmed with the substantial patient influx at the public hospital. Therefore, they suggested that the implementation of a new TM program would require the recruitment of separate staff to support TM program functioning:

Over here one thing is, that the patient burden is too much. So, maybe the doctor would not be able to see the daily readings or the data, which is received of the woman, or maybe she [the doctor] could not directly get in touch with the patient. Yes, but if there is someone specific [Clinician or Nurse], who will be overseeing these patients then that would be easier as they will have to see only those patients, who are already booked with them and with whom they would be doing telemedicine or this TM.

Clinician 04

Key informants believed that the senior health care providers who are accustomed to seeing patients in clinics might resist change and show reluctance in learning new technology. A clinician highlighted that the health care providers who are comfortable in seeing patients during in-person visits might believe that the use of the TM program may cause issues in identifying important signs that could be monitored during physical examination:

Because they are so much used to the manual part and they are so much used to the physical presence of the patient that they will be reluctant to use new technology...they are used to seeing patients in their clinics; thus, they will think that the TM may miss the physical examination sorts of things of the patient.

Clinician 04

Regarding willingness for TM program use among pregnant women, most pregnant women stated desire and trust in using the TM program to share their blood pressure readings with their providers regularly, whereas a few pregnant women were unable to recognize the usefulness of the TM program and showed a lack of willingness to use it because of anticipated trust issues and harms associated with the use of the TM program. Pregnant women were particularly concerned about the accuracy of blood pressure readings as the blood pressures would be measured using electric machines:

The disadvantage can be this that it is an electric thing and there is no reliability of it. It can give wrong readings...even if you take a new blood pressure machine.

Woman with pregnancy 06

A few pregnant women showed a lack of willingness to use the TM program as they were concerned that blood pressure machines and smartphones might emit radiation that could be harmful to their fetus:

It [TM program] is a bit risky because the child might get rays...like the usage of microwaves.

Woman with pregnancy 05

To address trust issues and safety concerns among pregnant women, key informants suggested that it is necessary to fully understand the needs and concerns of patients and providers before implementing a TM program. Furthermore, key informants suggested the provision of incentives to pregnant women and health care providers to promote and increase TM program use, such as paying for transportation costs:

In my opinion, in this, you shall need to give some incentive to pregnant women and providers at some stage. You need to give them some support to kick-start the project...Like the women be given some incentive like you place some transportation cost.

Digital health expert 13

#### Weak Technological Literacy to Use TM Program

The interviews also revealed concerns regarding the technological literacy of pregnant women and health care providers in using the TM program. Key informants believed that senior health care providers might find it difficult to use the TM program, whereas many young health care providers may find it easy to use. A digital health expert mentioned that most health care providers usually wish to continue their regular conventional clinical practices and stay limited in terms of the scope of their work:

I think it is mainly the interests that they would have in such kind of a thing, many health providers here want to stay limited in what they are doing, they just want to do regular practice and that is it.

Digital health expert 09

All study participants unanimously believed that the young, educated, and financially secure pregnant women would be more able to learn and use the TM program as opposed to older women who are uneducated and belong to a low socioeconomic class. Nonetheless, it was mentioned that such women would be able to receive help from immediate family members to participate in a TM program:

Those who would be educated would be able to use it, and those who would not be educated would not be able to use it.

Woman with pregnancy 01

To address concerns related to technological illiteracy, key informants and pregnant women suggested building the capacity of health care providers and pregnant women through adequate training and demonstrations. In addition, key informants and pregnant women highlighted the need to train people in support networks such as husbands, in-laws, daughters, and sisters on the use of the TM program to increase TM adoption among older women:

I mean what you have told me right now, theoretically, the idea sounds doable, what resources it involves I do not really know, but one important factor that needs to be kept in mind is that women and their family members have to be trained and you need to make sure that they do it the right way.

Digital health expert 12

#### Lack of Technological Infrastructure for Implementation of TM Program

Key informants mentioned that the weak technological infrastructure, including poor access to smartphones, the internet, and blood pressure machines, might hinder TM program implementation. On the other hand, some key informants verbalized that cell phone penetration has increased significantly as a growing number of pregnant women coming to public hospitals have either personal or shared access to basic mobile phones. To address concerns related to weak technological infrastructure, digital health experts highlighted the need to use basic mobile phones and their functions such as SMS text messaging and phone calls to engage and empower more women for TM program use. A digital health expert highlighted the importance of using the Global System for Mobile Communications to leverage basic mobile phones’ SMS text messaging function, given that the vast majority have access to basic mobile phones:

I think that GSM is still better. There are many people in our communities, who are still dependent on non-smartphones and that is where GSM plays a very important role. So, if that data can very simply be entered or directly transferred from the machine to your GSM device, I would say that is a better way. This is what we did in our monitor that the data is connected, and it is sent through the text messaging to their own phones and to the phones of health providers rather than using any kind of internet because that is another limitation. Because I doubt, the most that they would be able to use the Bluetooth-enabled technologies and smartphones themselves. We have to make sure that it is very easy to use, very simple.

Digital health expert 09

#### Sociocultural Factors Affecting Program Use

When asked about anticipated sociocultural factors influencing TM program use, most women expressed that they might face restrictions on the use of the TM program from mothers-in-law and husbands. Most women mentioned that they would require permission from their husbands and in-laws before opting into the TM program. This perception was echoed by some key informants, who also thought that family members might discourage pregnant women from using the TM program and buying blood pressure machines. An MNCH specialist expressed that women are not allowed to use phones because of cultural reasons, and, therefore, these women can be reached through their husbands’ phones:

We are getting some families who do not allow us to use phones because of cultural barriers. They [pregnant women] are not allowed to keep the mobile phones or use the internet and you know, messaging and everything on phones. So, their husbands had the phones, and these women are contacted through their husband’s phones. The women are not directly allowed to keep the phone. So, this is a challenge too, the cultural one.

MNCH specialist 10

However, other pregnant women at high risk for pre-eclampsia and key informants verbalized that, in most cases, there would be no cultural restrictions, and women would be able to receive support from their immediate family members for TM program use. A digital health expert mentioned that it is only an assumption that the TM program will not be accepted in the community, and there will be cultural restrictions on the use of the TM program:

I am saying that it is an assumption that people are not going to acknowledge it in the communities, my experience is that they are extremely receptive if the person who is giving that technology is known to them. So, the nurse is familiar to them they will be excited, they will be interested. Everyone wants to try new gadgets it is a human phenomenon, and they are no different. I do not see a cultural barrier in trying a new instrument.

Digital health expert 02

To respond to sociocultural factors associated with the use of the TM program, pregnant women and key informants, including clinicians, nurses, and digital health experts, suggested sensitizing the community and family members to encourage TM program use for supporting pregnant women at high risk. Digital health experts suggested having a team to work around the sensitization component. The main component of sensitization and advocacy was deemed to be the involvement of families in the decision-making process at the very onset of recruitment into the program for improving the uptake of the TM program among pregnant women as family members would be made responsible for sending the information to providers:

As I said that whatever these sorts of things need to be initiated. If at the first go it should be like the decision including the family, then acceptance will be very good. But if we just tell the woman and she goes back and tells, then I do not think there will be acceptance. They [family] may think that they are using machines maybe it will impact their privacy. And these sorts of things. So, I always include one of the family members and make them responsible, instead of the pregnant woman. So, that works.

Clinician 04

### Considerations for Implementing the TM Program

#### Features and Ease of Use of TM Program

Clinicians and pregnant women suggested that the TM program should be designed in the local languages (Urdu or Sindhi), keeping in mind that health care providers, pregnant women, and people from their support network are proficient in at least one of these two languages. In addition, pregnant women and clinicians suggested a range of relevant maternal and fetal parameters to be included in the TM program, including the weight, hemoglobin level, complete blood count, calcium and vitamin D levels, and pre-eclampsia disease symptoms of pregnant women, as well as fetus weight and heartbeat. Some digital health experts, clinicians, and a few pregnant women suggested establishing an algorithm in the TM program, which could serve as a reminder and warning sign alert system to empower pregnant women, facilitate the patient’s role in decision-making, and serve as a real-time clinical decision support system for health care providers:

And I do not know how this device or instrument would look like or how it will monitor and give the results but if it is in the form of color...maybe it can show red, yellow, and green color bars. And that actually shows the mother, like she is okay, she is a bit in the not okay or maybe in danger.

MNCH specialist 03

Pregnant women and digital health experts emphasized the inclusion of an educational component in the TM program to address the issue of poor awareness and knowledge about disease conditions and their management. A digital health expert stressed that the educational component should serve the purpose of educating pregnant women through SMS text messages even if the device monitoring is stopped after the intervention period.

#### Handling, Maintenance, and Sustainability of TM Program

When asked about handling and maintenance of the TM program, pregnant women assured that blood pressure machines would be gently handled and safely stored. However, clinicians and nurses anticipated issues with the handling and maintenance of the machines. Clinicians and nurses at JPMC expressed that there might be issues with retrieving smartphones and blood pressure machines from pregnant women because of the high loss to follow-up rate among pregnant women. A clinician iterated that even if the equipment is retrieved, it may get damaged because of poor handling by pregnant women and family members. Through past experiences, JPMC nurses and clinicians suggested either charging a minimum fee for equipment use or keeping their official documents such as national identity cards and marriage certificates until equipment is returned to create accountability on the part of pregnant women:

There should be something that can guarantee the equipment return from the patient. I would suggest keeping an ID card or marriage certificate.

Clinician 01

Key informants suggested that the sustainability of the TM program would require a substantial commitment from the public health department for integrating the TM program into existing workflows. A key informant suggested developing a sustainability plan with associated documentation at the provincial level to ensure TM program sustainability through the fiscal year budget:

I think that monitoring would also be extremely important. I mean there is always the risk when you do such programs that the devices do not reach the right people. If it is made part of the system, right from the planners and their commitment down to the health providers, and the supervision level to make sure that these devices are used properly and goes to the right people.

Digital health expert 09

## Discussion

### Principal Findings

This study provides an in-depth investigation into the perspectives, needs, and preferences of a mobile phone-based TM program for pregnant women at high risk for pre-eclampsia in Karachi, Pakistan. The interviewed pregnant women at high risk, nurses, clinicians, MNCH specialists, and digital health experts perceived an opportunity to establish a TM program for early identification of pregnancy complications and prompt treatment, care continuity, self-management, and clinical decision-making. Although some providers thought that there were potential benefits of implementing a TM program, there was no consensus on whether it would increase or decrease the clinical workload. The study identified the need for having a dedicated clinician to help with the operationalization of the TM program. A cost–benefit evaluation could help determine whether the recruitment of additional staff is justified to support the implementation of a TM program. Pregnant women stated their willingness to use the TM program and thought that TM would be convenient and cost-effective and provide a sense of empowerment in their own health care. However, some pregnant women at high risk were apprehensive of TM program malfunctioning and safety concerns associated with the use of smartphones and automated blood pressure machines. The study data indicated varied capacity to learn and use new TM programs among pregnant women because of sociocultural and financial factors and technological illiteracy. The study identified the need to provide technological support for pregnant women and providers and sensitize the community and family members to address sociocultural barriers. Pregnant women and clinicians suggested the establishment of a warning sign alert system as part of the TM program and the inclusion of some maternal and fetal parameters to be monitored in the TM program. To ensure that TM equipment would be returned, the key informants suggested creating accountability on the part of pregnant women by charging a minimum fee for equipment use.

### Comparison With Previous Research

Previous research has informed the needs for TM programs, mainly in high-income countries [10,34-37]. This study was the first to provide unique insights into the needs of the TM program for pregnant women at high risk in an LMIC such as Pakistan. Our study supported some findings from previous studies conducted in high-income countries regarding the convenience associated with TM. For instance, the study by Van Den Heuvel et al [10] conducted in the Netherlands reported that compared with the experiences of hospital admission in high-risk pregnancy, TM allowed pregnant women to be in a comforting and private environment during an anxious time in their lives. Consistent with the perceptions voiced in our study, another study by Van Den Heuvel et al [34] on TM for complicated pregnancies in the Netherlands highlighted the advantages of monitoring from home, such as reduced stress, increased rest periods for patients, reduction of admission, and possible reduction of costs. Similar to our study, the Primer and Provider Selection Guide on telehealth (2013), a whitepaper developed by the LeadingAge Center for Aging Services Technologies in a high-income country, highlighted that TM is a useful tool for empowering pregnant women at high risk by encouraging them to identify and report symptoms of exacerbation of their condition [35]. In addition, consistent with our study, the Primer and Provider Selection Guide on telehealth also recognized the value of TM in terms of supporting clinician decision-making and providing prompt treatment [35].

Our study reported some caveats to the willingness of pregnant women living in LMICs to use the TM program, such as TM program malfunctioning and safety concerns. Consistent with our findings, the Primer and Provider Selection Guide on telehealth emphasized the use of safe monitoring technologies to provide an enhanced sense of security, prolonged independence, and improved quality of life [35]. In our study, providers indicated reservations in using TM because of the anticipated increase in workload associated with responding to alerts generated through the TM program. This is consistent with the study by Anderson et al [35] on *unpacking TM work,* which confirmed that TM is time consuming and considered a burden on the clinical workload [36]. Their study highlighted that telephone calls have an important function in TM, such as supporting clinical decision-making and enabling the provision of patient-centric care. However, the telephone calls between patients and clinicians were found to increase the time spent in remote monitoring [36].

Digital health experts in our study highlighted the need to use basic mobile phones and SMS text messaging functions to address issues associated with weak technological infrastructure. The Centers for Disease Control and Prevention (health protection agency in the United States) Guide (2019) on *using technologies for data collection and management* also recommends that the choice of technology platforms should be driven by the existing technological infrastructure, goals of the investigation, and training and skills of available staff [37]. To address the issue of technological literacy for TM program use, our study findings suggested building the capacity of pregnant women and health care providers through training and demonstrations. This is consistent with the Primer and Provider Selection Guide, which emphasized the value of clearly defining the new model of care and preparing staff and end users through training and support plans before starting any new program [35].

In contrast to studies conducted in high-income countries, our study found some interesting findings on the sociocultural factors influencing TM program use in the LMIC context. Our study highlighted that pregnant women in LMICs might face restrictions from mothers-in-laws and husbands or require permission from them for using TM program. A similar finding was reported in the qualitative study by Qureshi et al [38] on health-seeking behaviors during pregnancy. The study concluded that maternal health care use is heavily influenced by social, economic, and cultural factors in rural Pakistani communities [38]. Their study revealed that principal decision makers for health care use are husbands and mothers-in-laws, and thus, women are expected to follow their decisions [38]. To respond to sociocultural factors associated with the use of the TM program in LMICs, our study identified the need to sensitize the community and family members on the usefulness of the TM program and to involve families in the decision-making process at the onset of recruitment into the TM program to help with its uptake.

### Recommendations

Our findings suggest that TM could be successfully designed, implemented, and sustained in an LMIC such as Pakistan to support the early identification of pregnancy complications and prompt treatment. We offer the following recommendations for clinicians, MNCH specialists, digital health experts, and policy makers to consider in developing a TM program to address the needs of pregnant women at high risk with pre-eclampsia and eclampsia in LMICs:

1. Building the capacity of pregnant women and health care providers through training and demonstrations could improve technological literacy for TM program use.
2. There is a need to sensitize the community and family members on the usefulness of the TM program to address sociocultural factors affecting TM program use. This could be done at the very onset of recruitment into the TM program to improve its uptake.
3. Clinicians and nurses are in a unique position to use the TM program; however, this might increase their clinical workload associated with the TM of pregnant women. Therefore, an approach using user-centered design and phased implementation could help to determine the impact of TM program use on current clinical workload and establish the need for additional staff to ensure adequate adoption of the TM program.
4. Future work should consider greater levels of co-design and the engagement of consumer representatives to ensure the development of a context-specific TM program. For instance, given that the vast majority of the population in LMICs such as Pakistan have access to basic mobile phones, basic mobile phones and SMS text messaging functions instead of smartphones should be considered for use in a TM program.

### Strengths and Limitations

A strength of the study is the use of multiple data sources (pregnant women, digital health experts, MNCH specialists, clinicians, and health care providers), which assisted researchers in data triangulation and identification of converging and diverging lines of inquiry. Another strength was that the primary researcher maintained a reflexive journal during all stages of the research to recognize and acknowledge biases during the research process.

Limitations to the study included that it was conducted with a focus on TM within a single setting (JPMC), which could limit the transferability of the findings to other settings [39]. However, this study may provide insights into other similar public hospitals across Pakistan and in other LMICs that are interested in supporting pregnant women at high risk through TM. Second, caregivers were not allowed to be present during interviews with pregnant women because of the COVID-19 pandemic, which may have limited the exploration of shared narratives on the needs of the TM program. Third, the researchers were unable to conduct member checking with study participants as it would have been exceedingly difficult to contact patient participants after the initial interview. However, at the end of each interview, the primary researcher restated and summarized the information obtained during the interview with the aid of interview notes to ensure that the study data resonated with the participant’s perspectives and experiences. Finally, some participants were interviewed via the web through Zoom technology. Hence, the researchers did not have the opportunity to build rapport with the respondents or obtain nonverbal cues during interviews.

### Conclusions

The pregnant women at high risk for pre-eclampsia, clinicians, MNCH specialists, and digital health experts thought that a mobile phone–based TM program may be feasible to implement, largely as mobile phones are becoming increasingly pervasive in LMICs. The pregnant women and health care providers were willing to use a mobile phone–based TM program as they perceived many benefits, including early identification of pregnancy complications and prompt treatment, convenience, cost-effectiveness, increased sense of empowerment in their own health care, and improved care continuity. However, participants cited several caveats to their willingness to use the TM program: the monitoring system would have to be easy to use, the TM program must be safe, and the blood pressure measuring device must be reliable. Recommendations from this work include the following: (1) build the capacity of patients and providers on TM program use, (2) sensitize the community and family members on the usefulness of the TM program, (3) use an approach incorporating user-centered design and phased implementation to determine the clinical workload and establish the need for additional staff for TM program monitoring, and (4) ensure greater levels of co-design and the engagement of consumer representatives. The findings from this study highlight the perceived feasibility of a mobile phone–based TM program for pregnant women at high risk for pre-eclampsia and provide insights that can be directly used for the design of future TM programs with the aim of reducing mortality and morbidity from pre-eclampsia and eclampsia in LMICs. A future feasibility study will be conducted based on the findings of this study to determine if an effectiveness trial is warranted.

## Abbreviations

AKU: Aga Khan University
CLIP: Community-Level Interventions for Pre-eclampsia
JPMC: Jinnah Post Graduate Medical Center
LMIC: low- to middle-income country
MNCH: maternal, neonatal, and child health
OB-GYN: obstetrics and gynecology
TM: telemonitoring
